# Disenfectant

**Published:** 1856-10

**Authors:** 


DISENFECTANT.—One pint of the "Liquor of Chloride of Zinc" in one pailfull of water, and one pound of Chloride of Lime in another pailfull of water. This is perhaps the most effective, theoretically and practically, of anything that can be used, and when thrown into privy vaults, cesspools, or upon decaying matter of any description, will effectually destroy all offensive odors. The cost of these substances is thirty-five cents.

We know of a better one than this. Keep every spot of your dwelling scrupulously clean and dry, from cellar to garret, and from the line fence in the rear to the center of the street in front.

